# Association of ERCC gene polymorphism with osteosarcoma risk

**DOI:** 10.4314/ahs.v20i4.39

**Published:** 2020-12

**Authors:** Guanliang Wang, Jianping Li, Xiling Xu, Ramit Kumar Gupta, Xiaoqiang Gao

**Affiliations:** 1 Department of TCM, Army 75th Group Military Hospital of People's Liberation Army, Dali 671000, China; 2 Department of Surgery and Anaesthesia, the Affiliated Hospital of Putian University, Putian 351100, China; 3 Department of Pediatric cardiology, Guangzhou Children's and women's Medical Center, Jinan University, Guangzhou, 510632, China; 4 Department of Orthopedics, the Affiliated Hospital of Putian University, Putian 351100, China

**Keywords:** Osteosarcoma, overall survival of osteosarcoma, ERCC, gene polymorphism, meta-analysis

## Abstract

**Background:**

The relationship between ERCC gene polymorphism and osteosarcoma risk / overall survival of osteosarcoma is still conflicting, and this meta-analysis was performed to assess these associations.

**Material and methods:**

The association studies were identified from PubMed, and eligible reports were included and calculated using meta-analysis method.

**Results:**

Four studies were included for the association of ERCC gene polymorphism with osteosarcoma risk, and nine studies were recruited into this meta-analysis for the relationship between ERCC gene polymorphism and overall survival of osteosarcoma. The meta-analysis indicated that ERCC1 rs3212986 (8092 C>A) gene polymorphism, ERCC1 rs11615 (19007 T>C) gene polymorphism, ERCC2 rs1799793 (A>G) gene polymorphism, ERCC2 rs13181 (Lys751Gln) gene polymorphism were not associated with osteosarcoma risk. ERCC1 rs2298881 (C>A) gene polymorphism, ERCC1 rs3212986 (8092 C>A) gene polymorphism, ERCC1 rs11615 (19007 T>C) gene polymorphism, ERCC2 rs1799793 (Asp312Asn) gene polymorphism were not associated with overall survival of osteosarcoma. Interestingly, ERCC2 rs13181 A allele and GG genotype were associated with overall survival of osteosarcoma, but AA genotype not (A allele: OR = 0.78, 95% CI: 0.65–0.93, P = 0.007; GG genotype: OR = 1.32, 95% CI: 1.05–1.65, P = 0.02; AA genotype: OR = 0.69, 95% CI: 0.45–1.04, P = 0.08).

**Conclusion:**

ERCC2 rs13181 A allele and GG genotype were associated with overall survival of osteosarcoma.

## Background

Human osteosarcoma, one of the most familiar forms of the primary malignant tumor to adolescents and adults, is a genetically heterogeneous bone malignancy with poor prognosis despite the employment of aggressive chemotherapy regimens 1–3. To develop a good indicator to predict the early diagnosis of osteosarcomas and to find a good indicator to predict the overall survival of osteosarcoma are urgently needed.

Within DNA repair genes, there lie a number of single nucleotide polymorphisms which may impair protein function and attenuate DNA repair capability, resulting in genomic instability and individual predisposition to malignancies 4. Excision repair cross-complementation (ERCC) gene encodes a protein that can play a rate-limiting role in nucleotide excision repair pathway^5^. Increasing attention has been drawn to the association of ERCC gene polymorphism with various types of human cancers. Current evidences show that ERCC gene polymorphism can take part in the pathogenesis of osteosarcomas and overall survival of osteosarcoma. This meta-analysis was performed to assess these associations.

## Methods

### Search strategy

The search was conducted in the databases of PubMed on May 1, 2019, and the relevant investigation were included. The retrieval strategy of “(ERCC2 OR ERCC3 OR ERCC1 OR excision repair cross-complementing) AND (osteosarcoma OR bone tumour OR bone cancer OR bone carcinoma) AND polymorphism” was entered into the PubMed database.

### Inclusion and Exclusion Criteria

Inclusion criteria: (1) The outcome must be osteosarcoma or overall survival of osteosarcoma; (2) The study included two comparison groups (case group vs control group); (3) report should give the data of ERCC genotype distribution.

Exclusion criteria: (1) Case reports, editorials and review articles; (2) Preliminary result not on ERCC gene polymorphism or osteosarcoma / overall survival of osteosarcoma; (3) Investigating the role ERCC gene expression to overall survival of osteosarcoma.

### Data extraction

The following information from each recruited investigation was extracted by two investigators independently (Guanliang Wang and Jianping Li): first author's surname, year of publication, ethnicity, control source of the control group and the number of cases and controls for ERCC genotypes. Frequencies of allele of ERCC were calculated for case group and control group. Quality assessment was assessed by Newcastle-Ottawa Scale (NOS) score, and it was regarded as a high quality (or low-bias risk) study when total stars achieved six to nine.

## Statistical analysis

Cochrane Review Manager Version 5 (Cochrane Library, UK) was used in this meta-analysis to count the extracted data from each report. The pooled statistic was counted using the fixed effects model. However, a random effects model was conducted when the P value of heterogeneity test was less than 0.1. Results were expressed using odds ratios (OR) for dichotomous data. 95% confidence intervals (CI) were also calculated. P < 0.05 was required for the pooled OR to be statistically significant, and I2 was used to test the heterogeneity among the included studies.

## Results

### Search results and reference quality assessment

**Figure 1:**
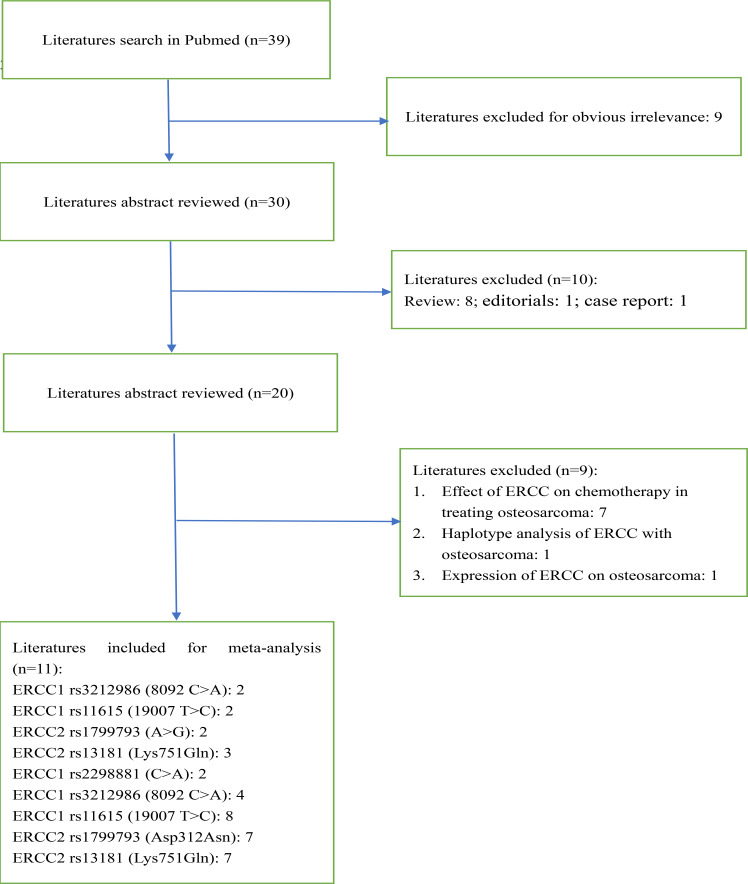
Flow diagram of the selection process

The database of Pubmed was searched for this meta-analysis, and 11 studies were eligible and included for this meta-analysis, and the recruited flowchart is shown in Figure 1. The NOS scores of all studies were more than 6, and the quality was regarded as a high quality.

### Association of ERCC1 rs3212986 (8092 C>A) gene polymorphism with osteosarcoma risk

Two studies 6,7 for the relationship between ERCC1 rs3212986 (8092 C>A) gene polymorphism and osteosarcoma risk were included in this meta-analysis. We found that ERCC1 rs3212986 (8092 C>A) gene polymorphism was not associated with osteosarcoma risk (A allele: OR = 0.79, 95% CI: 0.56–1.12, P = 0.18; AA genotype: OR = 0.68, 95% CI: 0.30–1.57, P = 0.37; CC genotype: OR = 1.05, 95% CI: 0.78–1.40, P = 0.76).

### Association of ERCC1 rs11615 (19007 T>C) gene polymorphism with osteosarcoma risk

Two studies 6,7 for the relationship between ERCC1 rs11615 (19007 T>C) gene polymorphism and osteosarcoma risk were included in this meta-analysis. We found that ERCC1 rs11615 (19007 T>C) gene polymorphism was not associated with osteosarcoma risk (C allele: OR = 1.17, 95% CI: 0.86–1.58, P = 0.32; CC genotype: OR = 1.16, 95% CI: 0.70–1.93, P = 0.58; TT genotype: OR = 0.85, 95% CI: 0.63–1.15, P = 0.29).

### Association of ERCC2 rs1799793 (A>G) gene polymorphism with osteosarcoma risk

Two studies 7,8 for the relationship between ERCC2 rs1799793 (A>G) gene polymorphism and osteosarcoma risk were included in this meta-analysis. We found that ERCC2 rs1799793 (A>G) gene polymorphism was not associated with osteosarcoma risk (A allele: OR = 0.85, 95% CI: 0.63–1.14, P = 0.28; AA genotype: OR = 0.82, 95% CI: 0.54–1.23, P = 0.33; GG genotype: OR = 0.86, 95% CI: 0.44–1.68, P = 0.66).

### Association of ERCC2 rs13181 (Lys751Gln) gene polymorphism with osteosarcoma risk

Three studies 7–9 for the relationship between ERCC2 rs13181 (Lys751Gln) gene polymorphism and osteosarcoma risk were included in this meta-analysis. We found that ERCC2 rs13181 (Lys751Gln) gene polymorphism was not associated with osteosarcoma risk (A allele: OR = 1.8, 95% CI: 0.93–1.78, P = 0.13; AA genotype: OR = 1.01, 95% CI: 0.72–1.43, P = 0.94; GG genotype: OR = 0.79, 95% CI: 0.53–1.17, P = 0.24).

### Association of ERCC1 rs2298881 (C>A) gene polymorphism with overall survival of osteosarcoma

Two studies 9,10 for the relationship between ERCC1 rs2298881 (C>A) gene polymorphism and overall survival of osteosarcoma were included in this meta-analysis. We found that ERCC1 rs2298881 (C>A) gene polymorphism was not associated with overall survival of osteosarcoma (A allele: OR = 1.06, 95% CI: 0.38–2.96, P = 0.09; AA genotype: OR = 0.64, 95% CI: 0.36–1.14, P = 0.13; CC genotype: OR = 0.60, 95% CI: 0.09–4.02, P = 0.59).

### Association of ERCC1 rs3212986 (8092 C>A) gene polymorphism with overall survival of osteosarcoma

**Figure 2:**
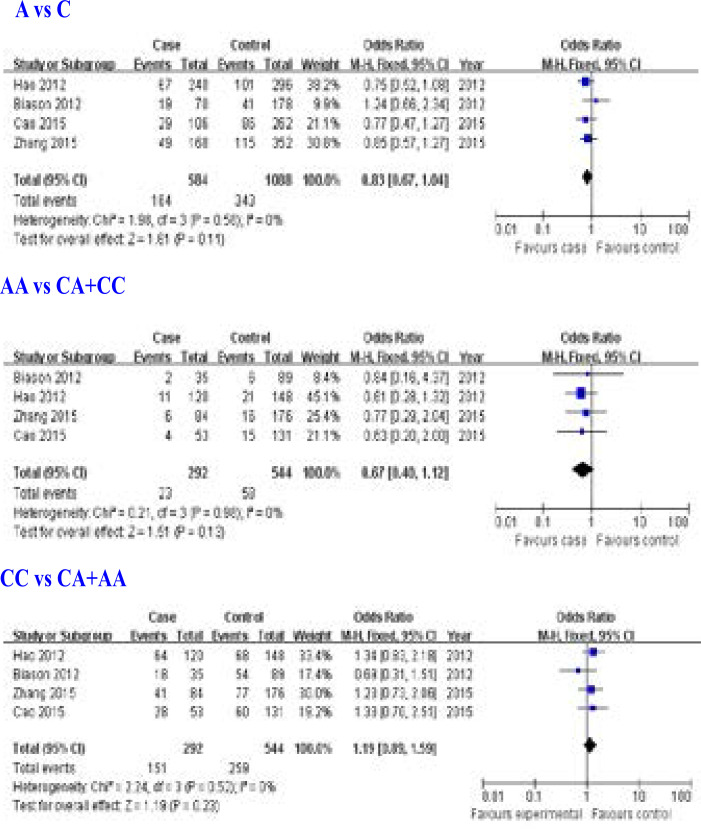
Association between ERCC1 rs3212986 (8092 C>A) gene polymorphism and overall survival of osteosarcoma

Four studies 6, 10–12 for the relationship between ERCC1 rs3212986 (8092 C>A) gene polymorphism and overall survival of osteosarcoma were included in this meta-analysis. We found that ERCC1 rs3212986 (8092 C>A) gene polymorphism was not associated with overall survival of osteosarcoma (A allele: OR = 0.83, 95% CI: 0.67–1.04, P = 0.11; AA genotype: OR = 0.67, 95% CI: 0.40–1.12, P = 0.13; CC genotype: OR = 1.19, 95% CI: 0.89–1.59, P = 0.23; Figure 2).

### Association of ERCC1 rs11615 (19007 T>C) gene polymorphism with overall survival of osteosarcoma

**Figure 3:**
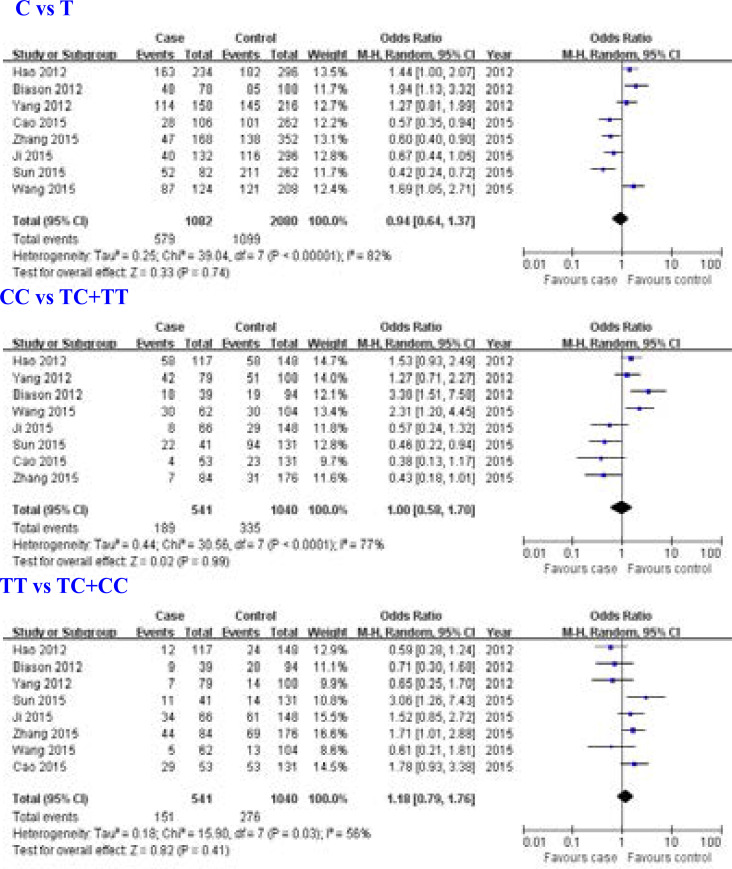
Association between ERCC1 rs11615 (19007 T>C) gene polymorphism and overall survival of osteosarcoma

Eight studies 6, 9–15 for the relationship between ERCC1 rs11615 (19007 T>C) gene polymorphism and overall survival of osteosarcoma were included in this meta-analysis. We found that ERCC1 rs11615 (19007 T>C) gene polymorphism was not associated with overall survival of osteosarcoma (C allele: OR = 0.94, 95% CI: 0.64–1.37, P = 0.74; CC genotype: OR = 1.00, 95% CI: 0.58–1.70, P = 0.99; TT genotype: OR = 1.18, 95% CI: 0.79–1.76, P = 0.41; Figure 3).

### Association of ERCC2 rs1799793 (Asp312Asn) gene polymorphism with overall survival of osteosarcoma

**Figure 4:**
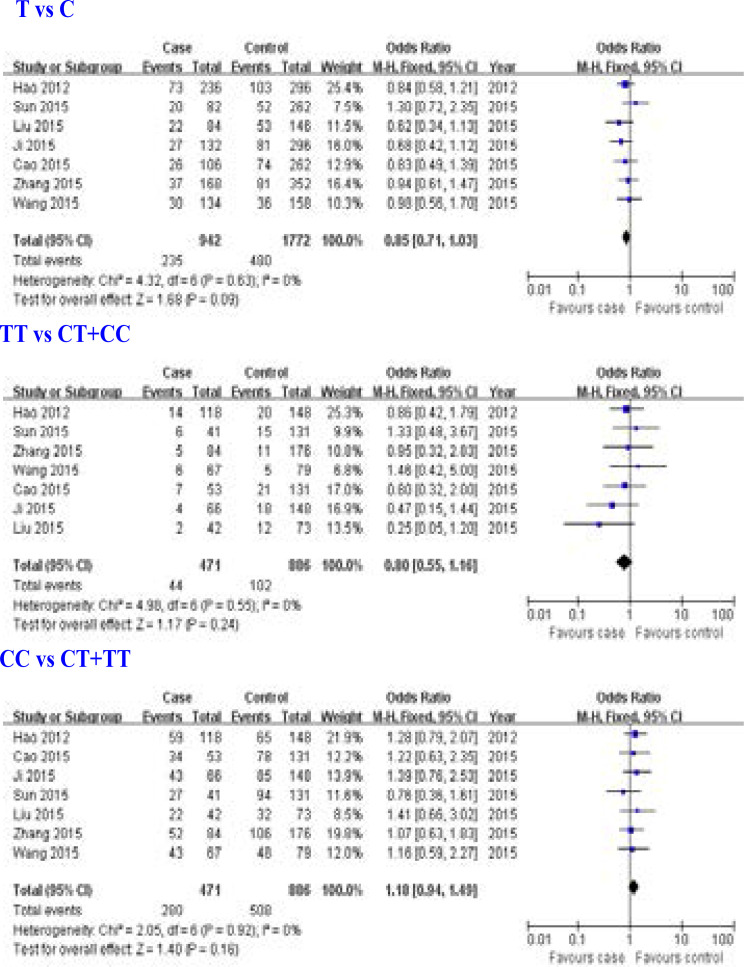
Association between ERCC2 rs1799793 (Asp312Asn) gene polymorphism and overall survival of osteosarcoma

Seven studies 9–12, 14–16 for the relationship between ERCC2 rs1799793 (Asp312Asn) gene polymorphism and overall survival of osteosarcoma were included in this meta-analysis. We found that ERCC2 rs1799793 (Asp312Asn) gene polymorphism was not associated with overall survival of osteosarcoma (T allele: OR = 0.85, 95% CI: 0.71–1.03, P = 0.09; TT genotype: OR = 0.80, 95% CI: 0.55–1.16, P = 0.24; CC genotype: OR = 1.18, 95% CI: 0.94–1.49, P = 0.16; Figure 4).

### Association of ERCC2 rs13181 (Lys751Gln) gene polymorphism with overall survival of osteosarcoma

**Figure 5:**
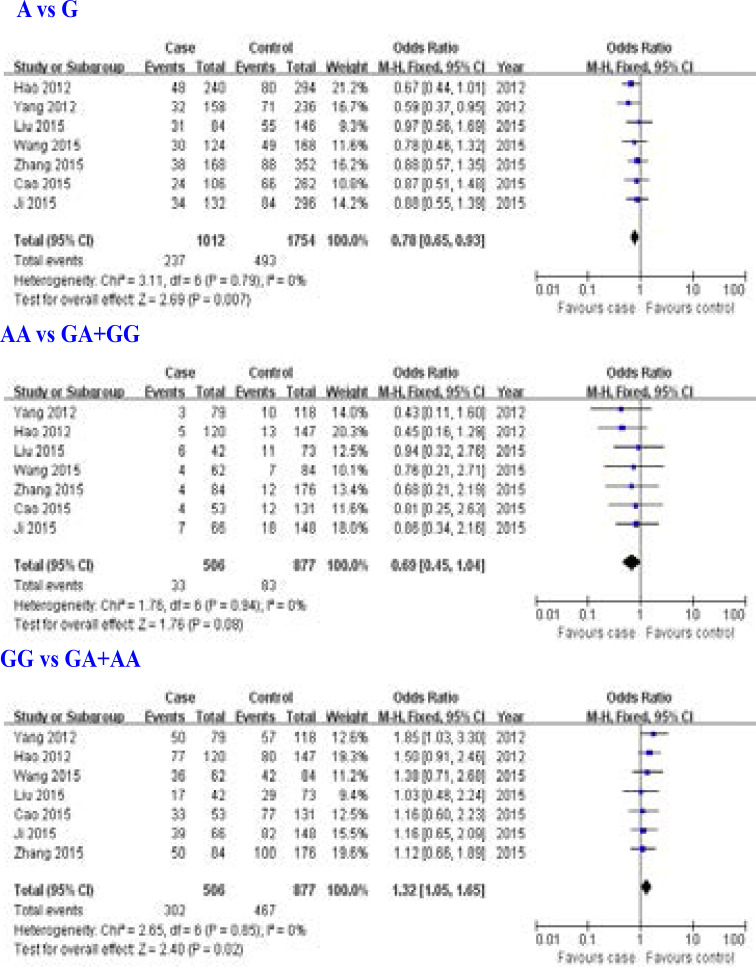
Association between ERCC2 rs13181 (Lys751Gln) gene polymorphism and overall survival of osteosarcoma

Seven studies 10–16 for the relationship between ERCC2 rs13181 (Lys751Gln) gene polymorphism and overall survival of osteosarcoma were included in this meta-analysis. We found that ERCC2 rs13181 A allele and GG genotype were associated with overall survival of osteosarcoma, but AA genotype not (A allele: OR = 0.78, 95% CI: 0.65–0.93, P = 0.007; G G genotype: OR = 1.32, 95% CI: 1.05–1.65, P = 0.02; AA genotype: OR = 0.69, 95% CI: 0.45–1.04, P = 0.08; Figure 5).

## Discussion

This meta-analysis was performed to detect the relationship between ERCC gene polymorphism and osteosarcomas risk, and the relationship between ERCC gene polymorphism and overall survival of osteosarcoma. We found that ERCC1 rs3212986 (8092 C>A) gene polymorphism, ERCC1 rs11615 (19007 T>C) gene polymorphism, ERCC2 rs1799793 (A>G) gene polymorphism, ERCC2 rs13181 (Lys751Gln) gene polymorphism were not associated with osteosarcoma risk. ERCC1 rs2298881 (C>A) gene polymorphism, ERCC1 rs3212986 (8092 C>A) gene polymorphism, ERCC1 rs11615 (19007 T>C) gene polymorphism, ERCC2 rs1799793 (Asp312Asn) gene polymorphism were not associated with overall survival of osteosarcoma. Interestingly, ERCC2 rs13181 A allele and GG genotype were associated with overall survival of osteosarcoma, but AA genotype not.

In previous, Li et al 17 performed a meta-analysis to assess the associations between ERCC polymorphisms and osteosarcoma prognosis by using meta-analysis, and reported that ERCC2 Lys751Gln was associated with the overall survival of osteosarcoma. In addition, there is no evidence of association on ERCC1 Asn118Asn, ERCC1 Gln504Lys, and ERCC2 Asp312Asn polymorphisms with prognosis in osteosarcoma. In our meta-analysis, we found that ERCC2 rs13181 A allele and GG genotype were associated with overall survival of osteosarcoma, but AA genotype not. The sample size in our meta-analysis was larger than the previous meta-analysis, and the results from our study might be more robust. In our meta-analysis, we also assessed the relationship between ERCC gene polymorphism and osteosarcomas risk, and reported that ERCC1 rs3212986 (8092 C>A) gene polymorphism, ERCC1 rs11615 (19007 T>C) gene polymorphism, ERCC2 rs1799793 (A>G) gene polymorphism, ERCC2 rs13181 (Lys-751Gln) gene polymorphism were not associated with osteosarcoma risk.

## Conclusion

ERCC2 rs13181 A allele and GG genotype were associated with overall survival of osteosarcoma. However, more association investigations are required to confirm these associations.

## Abbreviations

ERCC: excision repair cross-complementation; OR: odds ratios; CI: confidence intervals.

## Declarations

### Availability of data and materials

The datasets used and/or analyzed during the current study are available from the corresponding author on reasonable request.
